# Evaluating a 10-Week Family-Focused E-Health Healthy Lifestyle Program for School-Aged Children with Overweight or Obesity: A Randomized Controlled Trial Study Protocol

**DOI:** 10.3390/nu15132909

**Published:** 2023-06-27

**Authors:** Diana Zhu, Aimee L. Dordevic, Simone Gibson, Zoe E. Davidson

**Affiliations:** 1 Department of Nutrition Dietetics and Food, Monash University, Level 1, 264 Ferntree Gully Road, Notting Hill, Melbourne, VIC 3168, Australia; diana.zhu@monash.edu (D.Z.); aimee.dordevic@monash.edu (A.L.D.); 2 School of Clinical Sciences, Monash University, Level 5 Block E, Monash Medical Centre, Clayton, Melbourne, VIC 3168, Australia; simone.gibson@monash.edu

**Keywords:** e-health, overweight/obesity treatment, web-based, family-based

## Abstract

E-Health childhood obesity treatment interventions may serve as favorable alternatives to conventional face-to-face programs. More studies are needed to evaluate the effectiveness of such interventions beyond immediately post-program completion, including exploring program features impacting effectiveness. This randomized controlled trial with a qualitative component and waitlisted control group will evaluate the effectiveness of a 10-week family-focused e-Health program for school-aged children with overweight/obesity and explore the experience of families completing the program. The primary outcome is the change in BMI z-score and will be assessed from baseline to 10 weeks. Secondary outcomes include (the change in) waist circumference, dietary intake, physical activity, quality of life, and experiences, and will be assessed at baseline, post-10 weeks, and/or immediately, 3-, 6-, and/or 12-months post-program completion. Independent *t*-tests will be used to compare the differences in means and analyses of variances (ANOVAs) will be conducted to investigate the impact of the program or of being waitlisted and the effect size of the program on quantitative outcome measures. Reflexive thematic analysis will be used with qualitative data. Findings from this study are expected to provide learnings to upscale conventional childhood obesity treatment services, in the hopes of curbing the rising rate of childhood obesity.

## 1. Introduction

Obesity remains highly prevalent worldwide, with an increased prevalence of school-aged children (aged 5–16 years) living with severe obesity [1,2,3,4,5,6,7]. Children with overweight and obesity, particularly in their earlier childhood years, are also more likely to become adults living with overweight and obesity [1,2,3,4,5]. The increasing rate of childhood obesity influences increased healthcare and living costs for individuals globally [5]. For instance, in Australia, obesity is the second-largest contributor to health costs, estimated to cost Australia $11.8 billion, and if obesity rates continue to increase, it will cost an additional $87.7 billion in 10 years’ time [8]. 

The historically high global obesity rate over the past decade is attributable to the high accessibility to and the promotion of energy-dense foods, and technological advancements that encourage sedentary activities [4,9]. For instance, based on Australia’s 2011–2012 National Nutrition and Physical Activity Survey, less than 1 in 4 children aged 5–14 years engaged in the recommended duration of physical activity (at least 1 h of physical activity with moderate to vigorous intensity) per day, including children older than 10 years being more likely to engage in insufficient levels of physical activity per day compared to children younger than 10 years old [10]. The World Obesity Federation’s 2019 childhood obesity atlas also reported that at least 60% of children aged 10 years and older in the 191 included countries engaged in less than the recommended duration of physical activity per day [11]. In Australia alone, only about 1 in 10 children undertake the recommended amount of physical activity and sedentary behaviors (no more than 2 h of at-leisure screen-based activities) and consume the recommended serves of fruits and vegetables per day [10,12,13,14]. As a progressive, chronic condition impacting growth, development, and the onset of non-communicable, high-cost diseases, urgent action is needed to address obesity during school-aged years [1,2,3,4,9,15]. 

Internationally, countries recognize the importance of improving obesity treatment [16]. For instance, in 2022, Australia proposed a National Obesity Strategy, including an aim to reduce the prevalence of children living with overweight and obesity, and to ensure that Australians living with overweight and obesity have access to health care services [9]. Treating childhood obesity, including providing accessible and effective treatment programs, also supports progress in achieving United Nations’ sustainable development goals [17]. Studies suggest that multicomponent, family-based, individualized programs that encourage behavior change are most beneficial in the treatment of childhood overweight and obesity, and are recommended by the National Institute for Health and Care Excellence (NICE) and the American Psychological Association (APA) [2,3,4,9,18,19,20,21,22,23,24,25,26,27,28,29,30,31]. Such services, however, are largely lacking worldwide or are costly [15,31]. Existing multicomponent, family-based lifestyle programs with multidisciplinary specialist input are conventionally administered in-person and/or in group-based settings, with strict eligibility criteria, to a limited number of families at a time and, thus, are inaccessible to many children and their families [2,9,16,18,32]. Such services were designed to be implemented on a scale smaller than the growing number of families with children with obesity needing treatment. These programs also have long waiting lists or are costly [16,32]. For instance, a 2019 survey of multicomponent, multidisciplinary pediatric weight management clinics in Australia identified 17 services that are largely located in metropolitan cities and had waiting lists of 12 months or longer [2].

Digitizing such programs or offering electronic health (e-Health) programs, is a promising alternative overcoming the limitations of conventional programs. E-Health is the delivery of health-related information and/or healthcare services in a digital format or platform, and may serve as a standalone or adjunct intervention to conventional healthcare interventions, delivered face-to-face [3,22,33]. E-Health interventions have been shown to improve the accessibility, efficiency, and efficacy of healthcare services and people’s sense of accountability to manage their own health [3,18,20,21,22,23,24,25,26,29,33,34,35]. The ubiquitous use of the internet, including by populations of low socioeconomic status and/or remote areas, further supports the adaptation of such programs. In 2018, it was reported that more than 97% of Australian families have access to the internet using at least one mode of technology [28].

E-Health programs may serve as a favorable alternative to in-person programs, including for children. Children are avid users of the internet, including accessing the internet for health-related advice and services [3,23,27,28]. Research also suggests that younger generations may prefer receiving and/or accessing information on behavior change through digital avenues [3,22]. For instance, adolescents who received nutrition and physical activity education online were better able to impact positive lifestyle changes compared to receiving the program in-person [36]. With excessive screen time being particularly common among young individuals, as well as influencing the manifestation of childhood overweight and/or obesity, the implementation of e-Health services may serve as a strategy to leverage the time spent on devices to deliver treatment services. 

There is a developing body of evidence examining the effectiveness of e-Health interventions for the treatment of children with overweight and/or obesity. A 2021 meta-analysis of 19 randomized controlled trials (RCT) found that e-Health childhood obesity interventions significantly reduced BMI z-score (mean difference −0.063, Z = 4.66, *p* < 0.001) in children aged 3 to 16 years [3]. Similarly, a 2022 rapid review of 23 studies showed that family-based behavioral change e-Health programs in primary-school-aged children were able to significantly impact weight-based outcomes or lifestyle behaviors and/or habits [37]. This body of evidence, however, is largely limited to short-term program efficacy (i.e., immediately post-program with or without follow-up at 6 months or less), is parent- or child-focused as opposed to family-focused, and predominantly measures program effectiveness based on changes in weight-based outcomes [3,22,37]. These studies have also largely focused on e-Health programs delivered as adjunct interventions to conventional healthcare interventions delivered face-to-face. With the increased demands of healthcare services and drive for the delivery of remote services during the COVID-19 pandemic, more studies on interventions delivered predominantly via e-Health modalities vs. e-Health programs as adjuncts to conventional treatment services are needed to improve childhood obesity treatment programs.

Since the COVID-19 pandemic, healthcare systems have adopted online services and are committed to develop e-Health interventions for the prevention or treatment of diseases, including obesity [3,38]. The interest, preferences, and/or expectations of program end-users influence program uptake, use, and/or engagement; more studies exploring these outcome measures are needed, particularly to develop e-Health programs for the treatment of childhood obesity. As parents of school-aged children, particularly primary-school-aged children (aged 6–13 years), are recognized to greatly influence their child’s lifestyle behaviors and habits, exploring their perspectives of family-based lifestyle e-Health programs may inform program development [19,39]. Studies with this objective found that parents of early childhood and school-aged children, regardless of their socioeconomic status, prefer e-Health programs that involve the family unit, include evidence-based resources and interactive components, include individual support for families to set and achieve goals, focus on behavior change, are personalizable, and enable social support from other families completing the intervention [19,39]. Factors influencing a child’s engagement with a program may also differ from their parents’ perspectives; more studies exploring the child’s perspectives or experiences with e-Health interventions are, therefore, needed.

The online Better Health Program (BHPO) is a web-based program with health professional coaching sessions for the treatment of childhood overweight or obesity. The BHPO is the online adaptation of the in-person, group-based version, which is an evidence-based, family-centered lifestyle program for primary school aged children aged 7–13 years living with overweight or obesity, which has been successfully delivered and evaluated in Australia for over a decade. The in-person program is consistent with evidence-based guidelines on healthy eating, physical activity, and behavior changes [40]. An RCT evaluating the effectiveness of the face-to-face program demonstrated that it improved the degree of adiposity in children living with overweight or obesity, irrespective of whether families participated once or twice per week [40]. With changes related to the COVID-19 pandemic and digital culture encouraging a shift to e-Health modalities, the BHPO was developed. The web-based program is a 10-week multi-component e-Health program that involves weekly online modules/activity sessions educating on healthy eating, physical activity and behavior changes, weekly individualized phone calls with a health professional coach to support goal setting and program completion, the provision of supporting resources, and access to a closed Facebook group for the sharing of information and social support from other participating families. There has been no evaluation of the program in its online format. 

This protocol paper details the methodology of a study that aims to evaluate the effectiveness of a 10-week e-Health (web-based) healthy lifestyle program for primary-school-aged children aged 7–13 years (referred to hereon as “web-based program”). The primary aim of this study is to evaluate the effectiveness of a web-based program on the participating child’s health outcomes, including the impact of the web-based program on the child’s body mass index z-score (BMI z-score), waist circumference (WC), dietary intake, physical activity, and quality of life post-program-completion (immediately post-program, 6-months, 12-months). This study also aims to explore the experience of families completing a web-based lifestyle program.

## 2. Materials and Methods

The study protocol received ethics approval from the Monash University Ethics Committee (Project Number: 30472) and is registered with the Australian New Zealand Clinical Trial Registry (ACTRN12621001762842).

### 2.1. Study Setting

The intervention will run in Victoria, Australia, where the Better Health Program has not been delivered via e-Health modalities for the treatment of childhood overweight or obesity. All data collection and study/program activities will be undertaken via e-Health and with families living in Victoria. 

The obesity rate in Victoria is comparable to the obesity rate of developed countries with the highest rates of obesity, with 2 in 3 adults and 1 in 4 primary-school-aged children living with overweight or obesity [1,11]. 

### 2.2. Study Design

**Figure 1:**
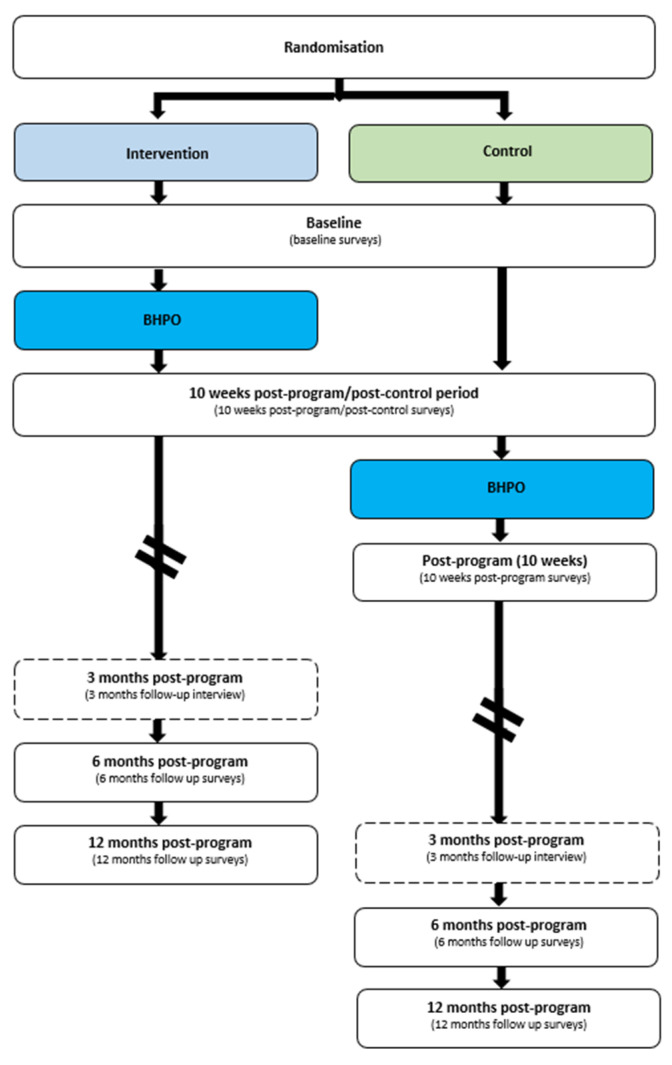
Randomized controlled trial with a waitlisted control group study design. Dashed box = optional post-program research follow-up activity.

This study is an RCT with a qualitative component and a waitlisted control group (Figure 1). This study is pragmatic in nature because it has been developed to evaluate the web-based program as it would typically be delivered. 

The 10-week web-based program will be administered by the administrating organization, Better Health Company (BHC), and is delivered during the year at primary school terms and midterms. In Australia, there are four school terms per year. The midterm programs will start approximately 6 weeks into the school term. Participants will be recruited for a school term or midterm program; a new recruitment cycle will begin, at the latest, 2 weeks prior to the commencement of the next earliest term or midterm program. Participants recruited during a school term or midterm recruitment cycle will only be considered for a school term or midterm program, respectively. Following study enrolment, participants allocated to the intervention group (Cohort 1) will complete the program in the subsequent commencing school term or midterm, whereas participants allocated to the control group (Cohort 2) will complete the waitlist control period in the next commencing school term or midterm. Participants allocated to the control group will not receive any intervention during the 10 weeks control period and will receive the (same) web-based program in the school term or midterm following the waitlist control term. Study involvement for each participant will run from baseline to 12-months post-program completion. 

Families who consent to the study will be randomized into the intervention or waitlisted control group. The randomization schedule was generated using block randomization (1:1 ratio) from Research Randomizer [41]. Group allocation will not be communicated to the BHC. Instead, study investigators will allocate participants to the respective school term or midterm in BHC’s data management system. In doing so, BHC’s health coaches, who are assigned to and have direct contact with families during the program, will be blinded to the study allocation.

### 2.3. Study Population

#### 2.3.1. Eligibility

This study/program focuses on the family unit. Study participants will, therefore, involve the child(ren) who meet the following inclusion criteria and at least one parent/caregiver. The eligibility criteria accounts for the inclusion criteria of the web-based program and the characteristics of participants who would benefit most from a family-centered program and are developmentally mature enough to complete research activities. 

##### Inclusion Criteria

The inclusion criteria are children aged 7–13 years (inclusive) with overweight or obesity, as defined by the Centers for Disease Control and Prevention (i.e., BMI ≥ 85th percentile), and living in Victoria, Australia [42]. Children will be generally healthy, defined as an absence of clinical or comorbid conditions that require medical clearance to participate. Children with a learning or behavioral disability will be included if their condition does not impact their ability to engage in the program. Children will also have at least one parent/caregiver who will accompany and support them with study/program activities and can provide informed consent. Children will meet all inclusion criteria of the web-based program as it would typically run, including being able to attend weekly coaching appointments scheduled on weekdays at 15:00–21:00. There are no other inclusion criteria for parents/caregivers.

##### Exclusion Criteria

Children will be excluded if they are within their healthy weight range or underweight (i.e., BMI 5th–84th or <5th percentile, respectively). Children will also be excluded if they are engaging in other weight management programs (structured or referred) and will not be discontinuing the program during the study time period. Children who have participated in or completed other program(s) delivered by BHC in the past 12 months will be excluded. Other exclusion criteria include having a sibling who is completing or already completed the study, being unable to understand or communicate in English, or lacking access to a device to access online resources and complete study/program activities.

#### 2.3.2. Sample Size Estimation

The primary outcome will be the change in BMI z-score from baseline to 10 weeks. An a priori power analysis was conducted using G*Power version 3.1.9.4 [43] to estimate the sample size needed to detect a difference in the mean change in BMI z-score between the intervention and control group from baseline to 10 weeks using a *t*-test. The sample size estimation accounted for a possible reduction in BMI z-score within the control group, an effect size based on BHC’s pilot data, and a 30% attrition rate based on previous trials on a similar program (trials on the MEND program reported a 30% attrition) [44]. The effect size based on the change in BMI z-score and informed by pilot data was 0.6 (i.e., medium effect size using Cohen’s (1988) [45] criteria). With a 5% significance and 80% power, this study aims to recruit *n* = 118 participants (*n* = 59 intervention group; *n* = 59 control group) to observe a difference in the primary outcome measure between groups with this effect size using a *t*-test. 

#### 2.3.3. Recruitment

BHC will recruit participants to complete the web-based program from their established networks in Victoria, including promoting the study among local government councils, schools, non-profit or private organizations, general practitioners, and other health professionals. BHC will advertise through flyers, posters, newsletters, social media posts (e.g., via Facebook, Instagram), and/or electronic correspondence. BHC will also use promotional strategies, including running family-focused media campaigns, keeping social media accounts up-to-date with recruitment information (e.g., promotional events, nearing the end of a recruitment phase), and reaching out to youth/parent-focused groups and/or health professionals in regional areas of Victoria.

Study investigators will also support recruitment by communicating with and disseminating promotional resources to professional networks.

Interested families can self-refer by completing an online registration form. Healthcare professionals may also refer participants by completing an online referral form. Once an online referral is received, a profile will be generated in BHC’s data system, including the participant’s unique identifier number. BHC will contact the families to outline the program and the relationship with the research, provide key information on the study, and conduct initial screening with participants for eligibility in the program and research. If families are willing to participate in this study, BHC will obtain verbal consent from the families for their contact details to be provided to study investigators. Study investigators will contact the families to address their queries or concerns if received and noted by BHC, confirm their eligibility for the research, and obtain written consent from the parent/caregiver and assent from the child(ren) prior to randomization.

### 2.4. Intervention

The BHPO is a 10-week web-based program designed for families that aims to promote healthy behavior changes. The program is a multi-component program, primarily delivered via a website where participants have access to weekly modules/activity sessions. There are 10 modules covering topics such as the five food groups, healthy eating behaviors/habits, reading food labels, the importance of and ways to increase movement, and healthy family routines. The participating child(ren) and at least one parent/caregiver will apply the information presented in each module by completing weekly online activities/exercises, available to families at the end of each module. Families participating in the program also receive weekly phone coaching calls with qualified health professionals such as dietitians, nutritionists, and exercise physiologists to set weekly targets on and develop action plans to achieve healthy behavior changes. Families also receive email/SMS communication to follow up with their progress throughout the program, access to a closed Facebook group with posts shared by other families who have previously participated or are currently participating in the program, and prize incentives upon completion. The program activities are designed to involve the family unit; the activities (e.g., learning and applying the information on healthy eating and physical activity, attending coaching sessions, making gradual behavior changes to meet weekly targets) are expected to be completed by the participating child(ren) and at least one parent/caregiver. The program is also designed and expected to allow the flexibility for household members other than the participating child and parent/caregiver to engage with the program contents (e.g., information, development and manifestation of action plans to meet targets/achieve healthy behavior changes). The program is delivered free of charge to participating families by BHC. The BHPO is adapted from the in-person, group-based version, which has been successfully delivered and evaluated in Australia for over a decade, and consistent with evidence-based guidelines on healthy eating, physical activity, and behavior changes [40].

No advice is provided to participants allocated to the control group during the waitlisted control period.

### 2.5. Data Management

Data collection and management follows the Monash Research Data Management Policy and data will be stored securely on Monash University supported servers and at the Monash University Be Active Sleep Eat (BASE) facility. All information collected by BHC at recruitment and during program administration will be stored in BHC’s online, password- and firewall-protected data system and will follow BHC’s privacy policy. All data and results will be deidentified and managed confidentially at all stages during the study.

Information on data collection and management is provided to participants (children and parents/caregivers) in the study explanatory statements (both parent/caregiver and child versions); these forms are emailed to parents/caregivers along with the study consent forms, and when study investigators are obtaining written consent from participants and prior to randomization.

### 2.6. Outcome Measures

Outcome measures will be self-reported via surveys administered online, at baseline, post-program completion (10 weeks), and 6- and 12-months post-program completion. Participants allocated to the control group will be asked to complete an additional set of surveys following the 10 week control period. All study assessments will be administered online via a Research Electronic Data Capture (REDCap) [46,47] link, Australian Eating Survey (AES) [48], or Youth Activity Profile (YAP) [49] link, and distributed to participants via email.

Qualitative data will also be collected online through video conferencing and real-time transcribing platforms (Zoom [50], Otter.ai [51]) at 3 months post-program completion.

All study assessments, including study surveys and interviews, will be completed by both the participating child and parent/caregiver. All outcome measures will be self-reported by participants to complement the virtual nature of the intervention and include the participation of families from all geographic locations of Victoria. A study by Chai and colleagues supports the use of parent-reported measurements that are collected online for web-based lifestyle programs [52]. The study found that Australian parents of primary-school-aged children were relatively accurate in reporting their child’s anthropometric measurements, including their weight and height [52]. 

The primary outcome measure is the change in the child’s BMI z-score. Secondary outcomes include (the change in) the child’s BMI z-score, waist circumference, dietary intake, physical activity, quality of life, and the participating child and parent/caregiver’s experience of completing the program. Details on the collection of the outcome measures are summarized in Table 1.

The web-based program also includes surveys to be completed by participants as a part of the program activities. Data collected from these surveys may supplement data collected by study investigators, to inform outcome measures. 

### 2.7. Data Analysis

All statistical analysis of quantitative data will be performed using IBM SPSS Statistics version 25 [46] and methods were confirmed with and reviewed by the Monash study statistician (T.P.). 

The primary focus of this study is to test whether the web-based program has an effect on BMI z-score over a 10-week period. An independent *t*-test will be used to compare the difference in the mean change in BMI z-score between Cohort 1 and 2 from baseline to 10 weeks. Independent *t*-tests will also be used to compare the differences in the quantitative outcome measures between cohorts from baseline to 10 weeks. A 2 × 2 mixed ANOVA will be conducted to investigate the impact of the web-based program (between factor: web-based program vs. control) on BMI z-score from baseline to 10 weeks (within factor). Separately, mixed model ANOVAs will also investigate the impact of the web-based program on the secondary outcome measures (i.e., WC, diet quality score, physical activity score, and quality of life scores) from baseline to 10 weeks. The impact of being waitlisted (Cohort 2) will be explored to ascertain whether receiving the web-based program following 10 weeks of waiting will differ on the quantitative outcome measures from Cohort 1 over time. If being waitlisted does not have an impact on the quantitative outcome measures (as anticipated), a one-way repeated measures ANOVA will be used to assess the effect size of the web-based program on quantitative outcome measures overtime with greater certainty (i.e., power). Independent *t*-tests may also be used to compare the differences in the mean changes in quantitative outcome measures between cohorts or compare the mean differences in quantitative outcome measures for all participants at immediately pre-program (baseline or post-10-weeks waitlisted period) and post-program completion. 

As the goal of including a qualitative component in this study is to understand lived experience, reflexive thematic analysis will be used to explore participants’ experiences of the web-based program [54,55]. Evaluation studies on similar e-Health programs largely lack the use of qualitative approaches; therefore, an inductive vs. a deductive coding approach [54] will be used and performed using NVivo [56]. The process of reflexive thematic analysis will include the following stages [54,55,57]: study investigators will read the transcripts of the interview sessions generated from the Zoom videoconferencing platform [50] to familiarize with the data. Study investigators will develop codes and a coding framework from the transcripts to code the transcripts thereafter. Study investigators will develop themes from the codes and coded data. Themes will be reviewed and further developed with the codes and coded data. The themes will then be grouped into broader categories to make sense of the data. The qualitative findings will add to the statistical findings of this study.

## 3. Discussion

Urgent action is needed to improve the treatment of childhood obesity. Few multidisciplinary weight management services currently exist for children outside the realm of primary health care, which, with limited resources, is predominantly focused on helping children with co-morbidities associated with weight and not the general population.

E-Health interventions are promising strategies for the treatment of childhood obesity. With modifiable risk factors, including engaging in excessive sedentary, digital-based activities, influencing the rising childhood obesity rates, family-focused e-Health treatment programs may serve as means to upscale conventional treatment interventions. Such programs may also adapt to the increased demands of healthcare services and (young) people’s way of living during the COVID-19 pandemic. 

Though there is a growing body of evidence suggesting the effectiveness of e-Health interventions for the treatment of childhood overweight and/or obesity, studies have largely focused on parent- or child-focused programs and short-term impacts on weight-based outcomes and/or lifestyle behaviors/habits [3,22,37]. Evidence on the specific components necessary for the successful uptake and effectiveness of family-focused e-Health treatment programs is also lacking [3,22]. This study’s methodology will add to the learnings of existing studies evaluating e-Health childhood obesity treatment programs, including addressing the aforementioned limitations [3,22,33]. This study also uses a strong design methodology; this study will use a waitlisted controlled, randomized trial, and is pragmatic in nature to deliver the intervention as it typically runs.

There are also strengths in the assessed intervention. This study involves a unique partnership between academics and industry to deliver a real-world family-focused e-Health intervention. The e-Health adaptation of the face-to-face, group-based program is expected to support family engagement; the web-based program includes elements expected from and favored by parents of children with overweight or obesity of (e-Health) childhood obesity treatment programs [19,39]. Program characteristics, including the web-based program’s mode of delivery, may also support program engagement from school-aged children with overweight or obesity themselves.

Future findings from this study are expected to add to the evidence regarding e-Health childhood obesity treatment interventions, including novel insights on ways to improve the treatment of childhood overweight and obesity. This study’s purpose also aligns with global commitments to achieve United Nations’ sustainable development goals. The results of this study will be published in peer-reviewed journals and presented at scientific meetings and conferences. No identifiable information will be disclosed.

## Figures and Tables

**Table 1:** Outcome measures and measurement methods.

Outcome Measure	Collection Method/Tool	Details
BMI z-score/change in BMI z-score	Calculated from height and weight	Participants will be asked to report the participating child’s standing height and weight, measured at home or by a health professional. Participants will be provided with a measurement kit, including a height ruler and measuring triangle and instructions on how to measure height and weight accurately (posted to participants from BHC following their initial calls) [52].
Waist circumference	Tape measure	Participants will be asked to report the participating child’s waist circumference, measured at home or by a health professional. Participants will be provided with a measurement kit, including a tape measure, and instructions on how to measure waist circumference accurately (posted to participants from BHC following their initial calls).
Dietary intake (diet quality score)	AES [48]	Participants will be asked to complete the AES, based on the participating child’s dietary intake. The AES is a validated food frequency questionnaire used to assess usual dietary intake of individuals aged 2 years and above [48].
Physical activity (physical activity score)	YAP [49]	Participants will be asked to complete the YAP, based on the participating child’s engagement in physical activity. The YAP is an online tool used to assess an individual’s physical activity, namely collecting information on the individual’s physical activity behavior/patterns to generate a physical activity score. The tool was developed in the United States and, for the purpose of this study, has been customized to reflect common word choices and expressions used in Australia [49].
Quality of life (quality of life score)	PedsQL [53]	Participants will be asked to complete the PedsQL (both the parent and child versions), based on the participating child’s quality of life. The PedsQL is a brief and validated questionnaire that assesses health-related quality of life in children and adolescents [53].
Experiences	Semi-structured interview via Zoom platform [50]	Families (the participating child and at least one parent/caregiver) will be invited to participate in a semi-structured interview at 3-months post-program completion. Families who consent to the interview will be invited to a videoconferencing meeting via the Zoom platform with a study investigator and asked to share their experiences of the BHPO, including their reflections on completing and any changes related to the program (during and after program completion). The interview will also be recorded and auto-transcribed using the Zoom platform [50].

AES: Australian Eating Survey, YAP: Youth Activity Profile.

## Data Availability

Data sharing is not applicable to this article as no new data were created or analyzed in this study at this stage.
